# Corticosteroid Prescribing Patterns in the Emergency Department for Acute Chronic Obstructive Pulmonary Disease Exacerbations: A Retrospective Analysis Following an Educational Intervention

**DOI:** 10.51894/001c.124542

**Published:** 2024-09-10

**Authors:** Christopher Nedzlek, Jacob Blanchett, Zachary Illg, Geoffrey DiGiacinto, Kathryn Cunningham, Samuel J. Wisniewski, Jacob Tuttle

**Affiliations:** 1 Department of Emergency Medicine Henry Ford Wyandotte Hospital, Wyandotte, MI, USA; 2 College of Osteopathic Medicine Statewide Campus System, East Lansing, MI, USA Michigan State University

**Keywords:** Corticosteroid, COPD, LOS, recommendations, steroid

## Abstract

**INTRODUCTION:**

COPD is a progressive lung disease with marked airflow limitation. It has a large global prevalence and is managed with antibiotics, bronchodilators, and corticosteroids. Despite the prevalence, corticosteroid prescribing regimens differ widely amongst providers. This study aims to evaluate baseline corticosteroid prescribing patterns, the ability to change corticosteroid prescribing patterns with the utilization of an educational initiative, and to evaluate the effect of corticosteroid dose on length of stay, 30-day hospital readmission, mortality, and total hospital insulin dosing.

**METHODS:**

This study was conducted via a retrospective observational study. Providers at a single institution answered a baseline questionnaire on COPD corticosteroid prescribing patterns and subsequently received an educational presentation regarding evidence-based corticosteroid recommendations. Data were then retrospectively obtained and analyzed evaluating corticosteroid prescribing patterns both pre- and post-educational intervention. Data were analyzed using IBM SPSS Version 25.

**RESULTS:**

The provider survey revealed that most (95.3%) administered 125 mg of methylprednisolone to patients treated for AECOPD. The most common reason a particular dose of corticosteroid was administered was due to previous teaching or practice patterns. The mean initial steroid dose of methylprednisolone decreased following the educational initiative from 114.24 mg to 72.8 mg (p < 0.01). This corresponded to a 69% (n=41) decrease of providers using 125 mg methylprednisolone (p < 0.01), and increased prescribing of 62.5 mg methylprednisolone by 42.6% (n=66). The mean LOS following hospital admission for AECOPD in the pre-intervention group was 5.80 days, while the mean LOS following the targeted educational intervention decreased to 4.82 days (p = 0.01).

**CONCLUSIONS:**

The implementation of an educational intervention may change provider corticosteroid prescribing patterns. Additionally, lower corticosteroid dose in the Emergency Department may decrease patient length of stay. Keywords: Corticosteroid, COPD, LOS, recommendations, steroid

## INTRODUCTION

Chronic obstructive pulmonary disease (COPD), also referred to as chronic bronchitis or emphysema, is a progressive lung disease characterized by largely irreversible airflow limitation,^1^ often associated with smoking.^2^ An estimated 384 million people suffer from COPD globally, and around three million people die annually making COPD the third leading cause of death worldwide.^3–5^ The Centers for Disease Control and Prevention (CDC) reported that in 2017 there were 923,000 emergency department visits with COPD and emphysema as the primary diagnoses.^6^

Acute exacerbations of COPD (AECOPD) are defined as sustained worsening of baseline dyspnea (i.e., difficulty breathing), often with associated cough and sputum changes (consistency, volume, color) warranting additional treatment.^7^ AECOPD are often precipitated by a multitude of factors including infection, smoking, and medication non-compliance. Common treatments for AECOPD include bronchodilators, corticosteroids, and antibiotics; the goal of these treatments for AECOPD is to minimize the current exacerbation’s symptoms and prevent subsequent morbidity and mortality.^8^

Systemic corticosteroids improve the forced expiratory volume in one second (FEV1), oxygenation, and shorten recovery time and hospitalization duration in AECOPD patients.^9^ Systemic corticosteroids have been the mainstay of therapy for patients with AECOPD following a 1999 randomized controlled trial conducted by the Veterans Administrative System which demonstrated that corticosteroids led to reduced treatment failure (defined as the need for mechanical ventilation, death, or intensification of drug therapy) compared to placebo.^10^ Subsequent studies have demonstrated that oral and intravenous corticosteroid administration are equally effective.^11^ However, the optimal dosing regimen (dose and schedule) for corticosteroids remains a debate point.^12^ As is typical with many drugs, corticosteroids are not without side-effects. Side effects of short-term corticosteroid use include hypertension, hyperglycemia, pancreatitis, and psychosis.^13^ Additionally, long-term use can have more deleterious effects including osteoporosis and adrenal insufficiency.^13^

Current guidelines recommend low-dose oral corticosteroids for the shortest duration possible. The Global Initiative for Chronic Obstructive Lung Disease (COLD) guidelines recommend a dose of 40 mg of prednisone daily for five days, with these recommendations primarily based on the REDUCE trial.^14,15^ Similarly, the 2019 National Institute for Health and Care Excellence guidelines recommend 30 mg of oral prednisolone daily for five days.^16^ The 2011 Joint American Thoracic Society and European Respiratory Society guideline recommends 40 mg of prednisone daily for 10-14 days.^17^

Despite the growing evidence to support low-dose corticosteroid use for AECOPD patients, there is a wide variation in corticosteroid prescribing patterns in the emergency department (ED).^14–17^ The goal of this study was multifactorial, first, to examine the individual practice patterns for prescribing corticosteroids for AECOPD among emergency medicine (EM) attending physicians, residents, and advanced practice providers (APPs). Secondly, to implement a corticosteroid educational intervention to attain a reduction in initial AECOPD corticosteroid dose. Lastly, to examine the impact of low dose corticosteroid prescribing on length of stay (LOS) following hospital admission, 30-day hospital readmission, mortality, and total hospital insulin dosing.

## METHODS

### Design

A retrospective observational study was designed to assess EM provider corticosteroid prescribing patterns. An educational initiative was conducted to provide evidence-based recommendations on proper corticosteroid dosing for AECOPD. The educational initiative was conducted through a PowerPoint presentation given at a monthly ED department meeting.^8^

Pre- and post-educational intervention provider corticosteroid prescribing data were then collected and compared to current evidence-based recommendations. Secondary endpoints, including LOS following hospital admission, 30-day hospital readmission, mortality, and total hospital insulin dosing (used as an indirect measure of hyperglycemia, a complication associated with corticosteroid were evaluated for patients requiring admission for AECOPD).^13^

The study was approved by the Henry Ford Health System Institutional Review Board (IRB) prior to data collection. Informed consent was waived as the study involved no more than minimal risk to the participants as determined by the IRB. Protection of human subjects remained a priority throughout the study as EM provider participation in the survey was voluntary and anonymous. Additionally, patient data obtained via chart review was immediately de-identified. Study data was stored on password-protected electronic storage devices and only accessible by study personnel. Study results were grouped and not linked to individual participants.

### Setting

This study and its participants were located at Henry Ford Wyandotte Hospital in Wyandotte, Michigan.

### Sample

The sample population for the study’s survey component consisted of EM providers and included residents, attendings, and APPs employed at Henry Ford Wyandotte Hospital. The sample population for the study’s chart review component consisted of patients who presented to the Henry Ford Wyandotte ED and subsequently required hospital admission for AECOPD. Inclusion criteria were all patients with a hospital admission and an International Classification of Disease, Tenth Edition (ICD-10) diagnosis code of J44.1 indicating AECOPD. Exclusion criteria were patients with a non-J44.1 ICD-10 diagnosis, and if the initial corticosteroid dose was not specified when administered prior to hospital arrival in the electronic medical record.

### Data Collection & Measurement

The survey consisted of six questions that examined the participants’ preferred AECOPD corticosteroid dosing and corresponding rationale. The participants included emergency medicine attending physicians, residents, and APPs. The survey was anonymous and self-administered online via Google Forms (Google, Mountain View, CA) and took approximately three minutes to complete. The survey was available to participants for completion during a four-week timeframe.

Pre-educational intervention data was collected for three months from January 2019-March 2019, with 152 individual patient charts reviewed. Post-educational intervention data was also collected for three months from January 2020-March 2020, with 155 patient charts examined. Pre- and post-educational intervention data were obtained via retrospective chart review. Patient data that was collected upon chart review included patient age, sex, previously diagnosed medical conditions, length of hospital stay following admission, amount of insulin used during patients’ hospitalization, and 30-day readmission rates.

### Statistical Analysis

Data was analyzed using IBM SPSS Version 25 by author SJW. T-tests were performed comparing the mean age, length of stay, initial steroid dose, and cumulative insulin dose pre- and post-educational intervention. Chi-square analysis was used to further analyze initial steroid dose as well as secondary variables of interest (gender, history of diabetes, 30-day readmit, and mortality) pre- and post-educational intervention.

## RESULTS

A total of 67 provider participants were identified as being eligible and were contacted by email for enrollment in the study. 64 provider participants completed the survey for a response rate of 94%. Data from the EM provider survey revealed that a majority of respondents (95.3%) administered 125 mg of methylprednisolone to patients being treated for AECOPD in the ED. The most common reason (73.4%) why a particular dose of corticosteroid was administered was due to previous teaching or the practice pattern in the ED. In comparison, only 2 (3.1%) of provider respondents based their dosing on evidence-based medicine. Additionally, less than one-third of provider respondents, 20 (31.3%), knew the equivalent oral dose of prednisone that 125 mg of intravenous (IV) methylprednisolone corresponded to. Table 1 shows the results of the survey.

**Table 1.** 

*Survey results (N=64)*	***n (%)***
**What dose of IV methylprednisolone do you routinely use in the treatment of patients with an acute exacerbation of COPD?**	
40 mg	1 (1.6)
60 mg	2 (3.1)
80 mg	0 (0)
125 mg	61 (95.3)
**Why do you use the above dose of methylprednisolone?**	
Evidence-based medicine	2 (3.1)
Previous teaching (practice pattern)	47 (73.4)
Ease of administration (medication formulation)	13 (20.3)
Other	2 (3.1)
**Oral corticosteroid use has been demonstrated to be equally as effective as intravenous use in treating patients with acute COPD exacerbation.**	
True*	59 (92.2)
False	5 (7.8)
**All the following are correct regarding the use of corticosteroids vs placebo in the treatment of acute COPD exacerbation, except:**	
Improves FEV1	3 (4.7)
Reduces hospital length of stay	3 (4.7)
Restores lung function more quickly	11 (17.2)
Causes hypoglycemia*	47 (73.4)
**What dose of prednisone does the Global Initiative for Chronic Obstructive Lung Disease (GOLD) report recommend for the initial treatment of acute COPD exacerbation?**	
20 mg for 5 days	2 (3.1)
40 mg for 5 days*	40 (62.5)
60mg for 5 days	22 (34.4)
80 mg for 5 days	0 (0)
**125 mg of methylprednisolone is roughly equivalent to what oral dose of prednisone?**	
80 mg	23 (35.9)
100 mg	7 (10.9)
125 mg	14 (21.9)
160 mg*	20 (31.3)

* Denotes correct answer

Patient data collected from the pre- and post-educational intervention periods were analyzed and means (i.e., averages) reported (Table 2). The mean age of patients in the pre- and post-intervention groups were similar, with an average age of 67.9 years (SD = 11.02) in the pre-intervention group and 66.9 years (SD = 11.63) in the post-intervention group. There was a decrease in mean LOS following hospital admission in AECOPD between the pre- and post-intervention group noting 5.80 days and 4.82 days, respectively (p=0.01). The mean initial corticosteroid dose (ISD) of methylprednisolone in the pre-intervention group was 114.24 mg (SD = 33.34), followed by a statistically significant (p < 0.01) reduction in ISD following the educational intervention to 72.8 mg (SD = 35.33). Finally, the mean insulin dose throughout a patient’s hospitalization differed between the pre- and post-intervention groups, with doses of 55.15 units and 34.96 units, respectively. However, this dose reduction failed to demonstrate statistical significance (p = 0.12). (Table 2)

**Table 2.** 

*Mean Pre- and Post-educational Intervention Data*						
	Pre-intervention (N=152)		Post-intervention (N=155)		p-value*	
Age (years)	67.9 (SD = 11.02)		66.9 (SD = 11.63)		0.49	
Length of stay (days)	5.80 (SD = 3.94)		4.82 (SD = 2.95)		**0.01**	
Initial steroid dose (mg)	114.24 (SD = 33.34)		72.8 (SD = 35.33)		**< 0.01**	
Cumulative insulin dose (units)	55.15 (SD = 124.77)		34.96 (SD = 98.50)		0.12	

* Independent t-tests performed

Next, pre- and post-intervention prescribing patterns for ISD at our community-based hospital were examined. Pre-educational intervention data revealed that 90.1% of providers prescribed an initial methylprednisolone dose of 125mg. Following the educational initiative, there was a 69% decrease in providers prescribing 125 mg methylprednisolone (p < 0.01). The most common ISD given post-initiative was 62.5 mg methylprednisolone, which increased by 0.01) and 40 mg methylprednisolone (+2.58%, p=0.12). (Table 3)

**Table 3.** 

*Pre and Post Descriptive Data for Initial Steroid Dosing*				
Initial steroid dose	Pre-intervention (N=152)	Post-intervention (N=155)	% change	p-value*
Methylprednisolone 125 mg	137 (90.13%)	41 (26.5%)	-63.6%	**< 0.01**
Methylprednisolone 62.5 mg	0 (0%)	66 (42.6%)	+42.6%	**< 0.01**
Methylprednisolone 60 mg	3 (1.97%)	30 (19.4%)	+17.4%	**< 0.01**
Methylprednisolone 40 mg	0 (0%)	4 (2.58%)	+2.58	0.12
**Other	12 (7.89%)	14 (9.04%)	N/A	N/A

* Chi-square analysis performed

**combined aggregate of statistically insignificant findings

The secondary variables of interest were evaluated and documented in Table 4. The pre-intervention group was found to have a statistically significant higher prevalence of diabetes history (39.1%) when compared to the post-intervention group (27.1%). No statistically significant differences were noted between the pre- and post-intervention groups when comparing patient data across sex, mortality, and 30-day readmissions.

**Table 4.** 

*Pre and Post Descriptive Data for Secondary Variables of Interest*				
	Pre-intervention (N=152)	Post-intervention (N=155)	p-value*	
**Gender**				
Male	90 (59.2%)	90 (58.1%)	0.91	
Female	62 (40.8%)	65 (41.9%)		
**History of diabetes**				
No	92 (60.5%)	113 (72.9%)	**0.03**	
Yes	60 (39.5%)	42 (27.1%)		
**30-day readmit**				
No	114 (75%)	130 (83.9%)	0.07	
Yes	38 (25%)	25 (16.1%)		
**Mortality**				
No	145 (95.4%)	151 (97.4%)	0.38	
Yes	7 (4.6%)	4 (2.6%)		

* Chi-square analysis performed

## DISCUSSION

Due to the lack of robust randomized controlled trials, there is a wide variation in corticosteroid administration and prescribing patterns for use in AECOPD. In our community emergency department, this variation in corticosteroid administration is readily observed with patients receiving anywhere from 40 mg of oral prednisone to 125 mg of IV methylprednisolone to treat AECOPD. (Table 3) Of the providers surveyed for this quality improvement/patient safety study, 61 (95.3%) of respondents used 125mg of methylprednisolone prior to the AECOPD educational intervention. The most common reason cited via survey for methylprednisolone dosing was previous teaching or practice pattern 41 (73.4%). On the other hand, the least common rationale with only 2 (3.1%) of the respondents indicated evidence-based medicine was the source of their corticosteroid dosing. Another contributing factor to the higher dose methylprednisolone may be the perceived ease of administration by nursing staff of the entire vial, as teamwork is essential to the functioning of a busy emergency department. Of the providers surveyed, 13 (20.3%) gave ease of administration as the reason for ordering their initial dose of corticosteroids. In our emergency department, methylprednisolone is supplied in single use vials of either 125mg or 40mg. The comparative ease of use of an oversized single use vial may contribute to 125mg of methylprednisolone being used most frequently in our emergency department.

One objective of this study was to evaluate the impact of an educational intervention on AECOPD corticosteroid prescribing patterns. The educational intervention focused on reviewing current evidence-based recommendations for corticosteroid use in AECOPD and asking each provider to consider utilizing the lower-dose protocol. We achieved a 63.6% reduction of 125mg methylprednisolone being given to patients with AECOPD. Without robust random clinical trials as well as the lack of prioritizing evidence-based medicine to indicate the best initial corticosteroid dose for treatment of AECOPD, most providers (73%) in our group deferred to previous teaching or their established practice pattern when determining their initial corticosteroid dose. Further, wide variation in corticosteroid administration and prescribing patterns for use in AECOPD persisted after the educational intervention. Of the providers that did decrease their initial corticosteroid dose, 66 (42.6%) ordered 62.5mg. This dose was believed to be utilized due to perceived ease in administration (1 ml or ½ of 125mg vial) and compatibility with nursing scanning in the EPIC. Initially, there was concern that a 60mg order would not be compatible with 125mg vial in EPIC. As such, 62.5mg methylprednisolone was suggested because it was unclear if lower doses (60mg, 40mg) would be compatible with RN EMR scanning. It was later determined that those lower doses were also compatible and could account for the 30 (19.4%) providers that ordered methylprednisolone 60mg. Of the remaining providers, 4 (2.6%) ordered 40mg, and 5 (3.2%) ordered prednisone 50mg orally.

In addition to altering practice habits to better align with national guidelines we also hoped to demonstrate positive downstream effects of a lower corticosteroid dose measured by length of stay following hospital admission, 30-day hospital readmission, mortality, and total hospital insulin dosing (used as an indirect measure of hyperglycemia). We did observe a decrease in patient length of stay with 5.80 (SD = 3.94) prior to the educational intervention vs. 4.82 (SD = 2.95, p = 0.01). This decreased hospital length of stay has important consequences such as decreased healthcare costs, hospital acquired infections, and other iatrogenic complications. Noting the importance of this finding, we appreciate the possibility of confounding variables. Thus, we believe that further research is warranted to evaluate LOS and corticosteroid prescribing further. Lastly, we were unable to demonstrate a total lower insulin dosing between the two groups which we postulate is due calculating the total insulin dose of patients instead of counting increased insulin requirements from baseline insulin dose of the patient. We believe altering the manner in which insulin was reported and calculated could have demonstrated fewer hyperglycemic events being observed in the post educational group.

### Limitations

Data collected from a single community-based institution may denote and propagate bias. At a single institution our patient population may differ from other locations regarding AECOPD severity or comorbidities. Our providers may also differ in general demographic and educational factors from other institutions. Additionally, as an educational institution with an active residency program, our population of providers may be more amendable to changing practice patterns.

## CONCLUSION

The implementation of educational interventions can change provider behavior to better align with best practice guidelines, as shown in this study. More specifically, in this study, educational intervention was used to change provider behavior regarding a lower dose corticosteroid protocol. The results demonstrate a reduction in the prescribing pattern of 125mg methylprednisolone by 63.3%. Further, using a lower corticosteroid dose we observed a decrease in AECOPD patients’ LOS from 5.80 days to 4.82 days (p < 0.01).

### Conflicts of Interest

None.
